# Association of mitochondrial DNA copy number with chronic kidney disease in older adults

**DOI:** 10.1186/s12877-023-04203-7

**Published:** 2023-08-24

**Authors:** Yang Liu, Ying Pan, Zijian Tian, Jing Wang, Fei Chen, Zhaoxu Geng, Qian Li, Ziqing Liu, Xiaozhou Zhou, Kaixin Zhou

**Affiliations:** 1 College of Life Sciences, University of Chinese Academy of Sciences, 100049 Beijing, China; 2 Department of Endocrinology, Kunshan Hospital Affiliated to Jiangsu University, 215300 Suzhou, China; 3 Institute of Biophysics, Chinese Academy of Sciences, 100101 Beijing, China; 4 Science for Life Laboratory, Department of Biomedical and Clinical Sciences (BKV), Linköping University, Linköping, 58183 Sweden; 5 Department of Endocrinology, Friendship Hospital, Beijing, 100029 China; 6 Guangzhou Laboratory, 510000 Guangzhou, China

**Keywords:** Biomarker, Mitochondrial DNA copy number, Chronic kidney disease, Older adults

## Abstract

**Background:**

Mitochondrial dysfunction in kidney cells has been implicated in the pathogenesis of chronic kidney disease (CKD). Estimation of mitochondrial DNA copy number (mtDNA-CN) is considered a convenient method for representing mitochondrial function in large samples. However, no study has investigated the association between mtDNA-CN and CKD in older adults with the highest prevalence. The objective is to examine cross-sectional and prospective associations between mtDNA-CN values and CKD risk in older adults to determine whether mtDNA-CN represents a novel potential biomarker for the recognition of CKD risk.

**Patients and methods:**

In a Chinese community-based cohort of over 65-year-olds, we included 14,467 participants (52.6% females). CKD was defined by eGFR < 60 mL/min/1.73 m^2^ or ICD-10 codes (patients = 3831 (26.5%)). Participants had peripheral blood levels of mtDNA-CN calculated from probe intensities of the Axiom CAS Array.

**Results:**

The risk of CKD prevalence decreased with mtDNA-CN per 1-SD increment, independent of established risk factors for older CKD (odds ratio [OR] per SD 0.90, 95% confidence interval [CI] 0.86, 0.93, *P* < 0.001), and has comparable strength of association with these established risk factors. Furthermore, the progression of kidney function was stratified according to the worsening of eGFR categories. The risk of kidney function progression to a more severe stage gradually decreased as the mtDNA-CN increased (*P* trend < 0.001). Non-CKD participants in the highest quartile of mtDNA-CN had a lower risk of developing CKD compared to the lowest quartile within 2 years of follow-up, reducing the risk of CKD by 36% (95% CI 0.42, 0.97; *P* = 0.037).

**Conclusions:**

Based on the analysis of the largest sample to date investigating the association between mtDNA-CN and CKD in older adults, higher levels of mtDNA-CN were found to be associated with a lower risk of CKD, suggesting that a reduced level of mtDNA-CN is a potential risk factor for CKD.

**Supplementary Information:**

The online version contains supplementary material available at 10.1186/s12877-023-04203-7.

## Introduction

Chronic kidney disease (CKD) is a progressive disease with no cure and high morbidity and mortality [1]. The prevalence of CKD is increasing rapidly due to rising risk factors such as diabetes, hypertension, unhealthy diet, inappropriate physical activity and metabolic syndrome, with older adults being at a higher risk [2]. However, the pathogenic mechanism is unclear and there are no obvious clinical symptoms until severe damage has occurred [3]. Therefore, the identification of reliable biomarkers is of utmost importance for early detection and prompt public health interventions that can help delay the progression of CKD [4].

Mitochondria are essential to proper kidney function since the kidneys are highly energy-demanding organs with abundant mitochondria [5]. Mitochondrial morphological changes and dysfunction are common features of kidney cells in both animal models and patients with CKD [6, 7]. However, the clinical impact of mitochondrial dysfunction in patients with CKD is poorly understood mainly due to the challenges in convenient and accurate evaluation of mitochondrial dysfunction [8].

Mitochondria has its own circular genome, with up to thousands of copies in each cell called mitochondrial DNA copy number (mtDNA-CN) [9]. Recently, mtDNA-CN has been proposed as indicative of mitochondrial function [10]. MtDNA-CN reflects the energy demand of a cell, and has a significant phenotypic impact by altering gene dosage [11]. Importantly, detection of mtDNA-CN in thousands of populations is getting easy and convenient with the advance in genomic technologies [12]. Moreover, there was previous research reported the mtDNA-CN measured in whole blood was significantly associated with global gene expression in 30 tissues, include kidney tissue, suggesting that blood-derived mtDNA-CN can reflect metabolic health across multiple tissues [13].

To date, observational studies have shown that a higher mtDNA-CN was associated with a lower prevalence of microalbuminuria which is an early sign of CKD [14]. Higher mtDNA-CN levels have also been associated with a lower risk of CKD incidence [15]. Furthermore, in a population of patients with CKD, higher mtDNA-CN has been linked to a lower risk of CKD progression, adverse clinical outcomes, and all-cause mortality [16–18]. However, these studies have primarily focused on middle-aged adults, and there is currently a lack of research exploring the relationship between mtDNA-CN and CKD risk in older adults. Given that older adults have the highest prevalence of CKD and undergo age-related physiological changes that can lead to a gradual decline in kidney function and an increased likelihood of comorbidities, older adults require more attention.

In this study we utilized health care and genomic data from a large population cohort of Chinese older adults. By estimating mtDNA-CN from peripheral blood samples, we first investigated the association between mtDNA-CN and CKD prevalence, and then examined its association with the progression of kidney function, as well as incident CKD.

## Materials and methods

### Study design and participants

This study was based on a large population cohort from the Kunshan county, Jiangsu Province, China, between May 2019 and August 2021, while they took the annual health examinations which were offered free to the local older adults. The residual blood samples from their health examinations were stored in a sample repository at -80 degrees Celsius. The DNA was extracted from the blood samples within the same year of sample collection for genotyping based on the CAS SNP array chip. The detailed procedure has been reported by our research group in a previous study [19].

Our inclusion criteria for participants are qualified mtDNA-CN detection, no kidney cancer, and having eGFR information. Finally, this study included 14,467 participants aged over 65 with health examination information obtained at the same time as DNA blood samples testing, for the cross-sectional study of mtDNA-CN and CKD prevalence. Of these participants, 7,500 did not have CKD and underwent their next health examination within the following 2 years, resulting for the prospective study of mtDNA-CN and incident CKD. Details of the protocol for the current study was approved by the institutional review board of the First People’s Hospital of Kunshan (IEC-C-007-A07-V3.0). The study was performed according to the guidelines of the Helsinki Declaration.

### Definition of diseases

**1). CKD and kidney function progression**: CKD was defined according to the Kidney Disease Improving Global Outcomes (KDIGO) [20] and eGFR was calculated using the Chronic Kidney Disease Epidemiology Collaboration (CKD-EPI) formula [21]. A diagnosis of CKD was defined either estimated glomerular filtration rate (eGFR) was < 60 mL/min/1.73 m^2^ or based on diagnosis record data using International Classification of Diseases-tenth version (ICD-10), as shown in Table S1 [22]. Kidney function progression defined by worsening of eGFR categories according to KDIGO guidelines: stage 1 (eGFR ≥ 90) stage 2 (eGFR 60–89), stage 3a (eGFR 45–59), stage 3b (eGFR 30–44), stage 4 (eGFR 15–29) and stage 5 (eGFR < 15), with stage 5 representing the most severe form.

**2). Comorbidities and clinical covariates**: Diagnoses of comorbidities (diabetes, hypertension, cardiovascular and cerebrovascular diseases) were obtained from health care data using ICD-10. Metabolic syndrome was defined based on five physical examination indicators, with a positive diagnosis requiring at least three of the five components [23].

### Measurement of mtDNA copy number

MtDNA-CN was estimated using the pipeline proposed based on CAS SNP Array, which was previously reported by this research group [19]. In brief, the mtDNA-CN was estimated by the intensity of fluorescent signal of mitochondrial markers indicating the copy number segments of mitochondrial DNA captured by the corresponding probes. The intensity of fluorescent signal were calculated by Log R Ratio (LRR) and adjusted for genomic waves according to GC content by PennCNV [12]. The final mtDNA-CN estimates were extracted from the mitochondrial LRR adjusted for autosomal LRR by Principal component analysis (PCA). The method of mtDNA-CN estimation was established and validated in previous study [19]. For the strength of association between mtDNA-CN estimated from SNP array and WGS, our correlation coefficient of 0.52 is comparable to the previous studies 0.33 ~ 0.72 [24, 25]. Furthermore, we applied a linear regression model to control for the potential confounding effects of age, sex, white blood cell counts, platelet counts, blood collection date, and batch variability. This enabled us to obtain standardized mtDNA-CN estimates, which were used for subsequent association analysis.

### Statistical analyses

Clinical characteristics were presented as mean (SD), median (IQR) and number (%) for continuous, abnormal and categorical variables respectively. ANOVA, Wilcoxon and chi-square tests were applied to evaluate differences between phenotypic variables in CKD/non-CKD group. Spearman correlation testing was applied to evaluate the correlation between phenotypic variables and mtDNA-CN. We investigated the association between mtDNA-CN and the risk of CKD prevalence using multivariate logistic regression, adjusting all statistically significant relevant phenotypes for age, sex, waist, systolic blood pressure, diastolic blood pressure, serum glutamic pyruvic transaminase, serum glutamic-oxaloacetic transaminase, total cholesterol, triglyceride, high-density lipoprotein cholesterol, low-density lipoprotein, fasting glucose and comorbidities. Then, we utilized Restricted cubic spline analysis in logistic regression model was used to derive the shape of relationship between mtDNA-CN and the risk of CKD prevalence. This analysis was conducted using the R programming package: smoothHR, survival, rms. The model had nodes at the 5th, 35th, 65th, and 95th percentiles of the distribution of mtDNA-CN. Sensitivity analyses were conducted for two diagnosed sources of CKD (CKD from ICD-10 or health examined kidney function of eGFR < 60 mL/min/1.73 m^2^). We also performed sensitivity analyses to explore the association of mtDNA-CN with CKD in different comorbidities. We used the same covariates in the cross-sectional analysis and in the prospective analysis.

Multinomial logistic regression was applied to analysis the association between mtDNA-CN and six stages of kidney function progression. Additionally, mtDNA-CN was categorized into quartiles among 7,500 non-CKD participants, and Cox proportional hazards models were used to estimate hazard ratios for the associations of mtDNA-CN quartiles with CKD incidence. Follow-up time was used as the time scale, with censoring at the time of loss to follow-up or end of follow-up period (August 2021). Statistical analyses were performed using R version 4.1, with significance defined as *P* < 0.05.

## Results

### Sample characteristics

Among the 14,467 participants included in the study, the mean age was 71.6 (5.7) years, 52.6% were women, 26.5% had CKD (Table 1). Compared with non-CKD participants, those with CKD had significantly lower mtDNA-CN (*P* < 0.001). Further, CKD patients in addition to having worse kidney function were found to be older, higher rates of women, higher SBP, TG, and SGOT, as well as higher prevalence of comorbidities. Conversely, CKD patients had lower levels of DBP and SGPT. It is noteworthy that the established risk factors of CKD not only exhibited significant difference between CKD and non-CKD groups in the participants, but also showed a strong correlation with mtDNA-CN (most Spearman’s *P* < 0.001).

**Table 1:** Analysis the baseline phenotypic variables difference in CKD/non-CKD and correlations with mtDNA-CN

Characteristics	Non-CKD	CKD	*P* value	mtDNA-CN^†^ Spearman’s *P* value
	N = 10,636	N = 3,831		
mtDNA-CN^*^	0.03 (1.00)	-0.08 (1.01)	< 0.001	—
**Demographic**				
Age (years)	69.0 [67.0, 73.0]	74.0 [70.0, 79.0]	< 0.001	0.99^‡^
Sex = female (%)	5332 (50.1)	2279 (59.5)	< 0.001	0.95^‡^
BMI (kg/m^2^)	24.2 [22.2, 26.4]	24.4 [22.4, 26.5]	0.001	0.29
Waist (cm)	86.0 [80.0, 92.0]	86.0 [80.0, 91.0]	0.81	0.002
**Laboratory**				
SBP (mmHg)	140.0 [129.0, 154.0]	142.0 [130.0, 155.0]	0.004	< 0.001
DBP (mmHg)	79.0 [72.0, 87.0]	78.0 [71.0, 85.0]	< 0.001	0.19
SGPT (U/L)	17.0 [13.0, 23.0]	17.0 [13.0, 23.0]	0.026	0.83
SGOT (U/L)	21.0 [18.0, 25.0]	22.0 [19.0, 27.0]	< 0.001	< 0.001
TC (mmol/L)	4.7 [4.1, 5.4]	4.8 [4.1, 5.5]	0.19	< 0.001
TG (mmol/L)	1.3 [1.0, 1.9]	1.4 [1.1, 2.0]	< 0.001	0.007
HDL-C (mmol/L)	1.3 [1.1, 1.5]	1.3 [1.1, 1.5]	0.024	< 0.001
LDL-C (mmol/L)	2.7 [2.2, 3.2]	2.7 [2.2, 3.3]	0.94	< 0.001
Fasting glucose (mmol/L)	5.4 [4.9, 6.2]	5.6 [5.1, 6.4]	< 0.001	< 0.001
Creatinine (µmol/L)	70.0 [61.0, 79.8]	87.0 [73.6, 106.0]	< 0.001	< 0.001
eGFR (mL/min/1.73 m^2^)	85.9 [76.8, 91.1]	61.8 [52.4, 75.1]	< 0.001	< 0.001
BUN (mmol/L)	5.5 [4.7, 6.5]	6.3 [5.2, 7.6]	< 0.001	0.016
Uric acid (µmol/L)	307.6 [257.0, 363.3]	349.1 [285.9, 422.2]	< 0.001	< 0.001
**Comorbidities**				
Diabetes = 1 (%)	2,149 (20.2)	1,023 (26.7)	< 0.001	< 0.001
Hypertension = 1 (%)	7,319 (68.8)	3,233 (84.4)	< 0.001	< 0.001
Metabolic syndrome = 1 (%)	2,219 (20.9)	902 (23.5)	0.001	< 0.001
CCVD = 1 (%)	578 (5.4)	447 (11.7)	< 0.001	0.004

BMI: body mass index; SBP: systolic blood pressure; DBP: diastolic blood pressure; eGFR: estimated glomerular filtration rate; SGPT: serum glutamic pyruvic transaminase; SGOT: serum glutamic-oxaloacetic transaminase; TC, total cholesterol; TG, triglyceride; HDL-C: high-density lipoprotein cholesterol; LDL-C: low-density lipoprotein; BUN: blood urea nitrogen; CCVD: Cardiovascular and cerebrovascular disease

Data presented as median and interquartile range or number (%), unless otherwise indicated

^*^ Mean and standard deviation

^†^ Spearman correlation testing of mtDNA-CN versus phenotypic variables

^‡^*P* value was not significant as mtDNA-CN was adjusted for age, sex

### Association of mtDNA-CN with CKD prevalence

**Fig. 1:**
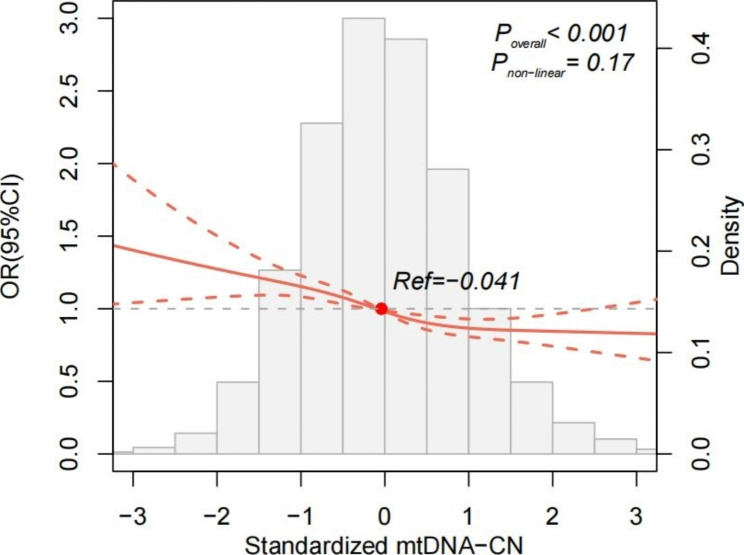
Association between mtDNA-CN and CKD prevalence based on restricted cubic spline function in the multivariate logistic regression model. The red line represented the adjusted OR corresponding to the different values of mtDNA-CN. The red dotted lines represented the 95%CI of the OR. The red closed circle, the reference value was set at the median − 0.041 for mtDNA-CN. The gray density histogram represented the frequency distribution of mtDNA-CN. The association of mtDNA-CN with prevalent CKD was significant (*P* value for correlation, < 0.001), the association was approximately linear (*P* value for nonlinear spline terms, > 0.05)

To determine the association between mtDNA-CN and CKD, we considered all phenotypes that could potentially confound the correlation. Logistic regression analysis showed that for each SD increment in mtDNA-CN, there was a 10% reduction in the risk of CKD prevalence (OR per SD 0.90, 95% CI 0.87, 0.93; *P* < 0.001, Table S2). The strength of this association was comparable to that of established risk factors such as fasting glucose, DBP and TG. After adjusting for all established risk factors, the Restricted cubic spline showed a significant linear dose-response association between mtDNA-CN and CKD prevalence (*P* overall < 0.001, *P* non-linear = 0.17, Fig. 1), suggesting that the odds of prevalence CKD decreased with mtDNA-CN.

Then, we conducted an analysis to investigate the association between mtDNA-CN and CKD in different comorbidity subgroups. Results were similar to those observed in the overall study population, we did not observe any interaction of comorbidities on the association between mtDNA-CN and CKD (Table S3). Furthermore, we performed pre-specified subgroup analyses by two sources of CKD diagnose, and tested subgroups of CKD for correlation with mtDNA-CN. Both subgroups observed a similar magnitude of association as with total participants (CKD_ICD_: OR per SD 0.88, 95% CI 0.81, 0.96; CKD_eGFR_: OR per SD 0.90, 95% CI 0.86, 0.94) (Table S4). This indicates that the correlation is not affected by the definition of CKD from different sources.

### Association of mtDNA-CN with kidney function progression

Kidney function progression was divided into six stages based on worsening of eGFR categories. We applied multinomial logistic regression models analysis between mtDNA-CN and each of the stage, the results showed the progress risk for kidney function to next stage was significantly decreased as mtDNA-CN per 1-SD increment (Table 2). Additionally, after adjusting for established risk factors, the higher mtDNA-CN was found to be significantly associated with a decrease risk of kidney function progression, which is consistent with the results of the univariate analysis (each stage versus stage 1, *P* < 0.01). Of note, the odds ratio decreases gradually with the increase of the mtDNA-CN, gradually reducing the severely risk of kidney function progression (*P* for trend < 0.001).

**Table 2:** Association of mtDNA-CN with kidney function progression

Kidney function	Number	Odds ratio (95% CI)		*P* value	*P* for trend
		Univariable	Multivariable		
stage1	3488	reference	reference	—	< 0.001
stage2	9200	0.96 (0.92,0.99)	0.94 (0.90,0.98)	0.0028	
stage3a	1299	0.87 (0.82,0.93)	0.84 (0.78,0.90)	< 0.001	
stage3b	367	0.70 (0.63,0.79)	0.66 (0.59,0.75)	< 0.001	
stage4	88	0.75 (0.61,0.94)	0.72 (0.58,0.91)	0.0051	
stage5	25	0.55 (0.40,0.77)	0.56 (0.39,0.79)	0.0013	

Multivariable model adjusted age, sex, BMI, waist, SBP, DBP, SGOT, SGPT, TC, TG, HDL-C, LDL-C, fasting glucose, diabetes, hypertension, metabolic syndrome, CCVD

### Association of mtDNA-CN with CKD incidence

During a median follow-up of 463 days in 7,500 participants without CKD, a total of 230 (3.1%) incident CKD cases were observed (Table S5). Cox regression analysis showed that mtDNA-CN was significantly associated with CKD incidence (HR per SD 0.85, 95% CI 0.72, 0.99; *P* = 0.038) (Table 3). However, this association was not significant after adjusting for established risk factors in the multivariate model (HR per SD 0.88, 95% CI 0.75, 1.03; *P* = 0.10). We further categorized mtDNA-CN into four quartiles based on the sample distribution and analyzed the associations of CKD incidence with different quartiles of mtDNA-CN. Participants in the highest mtDNA-CN quartile had a statistically significant lower risk of CKD incidence compared to those in the lowest quartile (*P* for trend = 0.12). The multivariable-adjusted results were consistent, showing a 36% reduction in the risk of CKD incidence (95% CI 0.42, 0.97, *P* = 0.037) in the highest quartile of mtDNA-CN.

**Table 3:** Hazard ratios for CKD incidence by levels of mtDNA-CN

mtDNA-CN	Events/n	Incidence Rate per 1000 Person-Yr	Hazard Ratio (95% CI)		*P* value
			Univariable	Multivariable	
mtDNA-CN^a^	230/7500	15.3	0.85 (0.72, 0.99)	0.88 (0.75, 1.03)	0.10
quartile1^†^(lowest)	59/1875	15.7	reference	reference	—
quartile2^†^	64/1875	17.1	1.09 (0.77, 1.56)	1.04 (0.73, 1.49)	0.82
quartile3^†^	71/1875	18.9	1.21 (0.85, 1.70)	1.18 (0.84, 1.67)	0.35
quartile4^†^(highest)	36/1875	9.6	0.60 (0.40, 0.91)	0.64 (0.42, 0.97)	0.037
*P* for trend					0.12

^*^ mtDNA-CN was continuous variable

^†^ mtDNA-CN was divided into four groups based on its quartile distributions in 7500 non-CKD participants

Multivariable model adjusted age, sex, BMI, waist, SBP, DBP, SGOT, SGPT, TC, TG, HDL-C, LDL-C, fasting glucose, diabetes, hypertension, metabolic syndrome, CCVD

## Discussion

In this large community-based cohort of Chinese old adults, we found that higher mtDNA-CN was associated with lower risk of CKD. The risk of kidney function progression significantly decreased as the mtDNA-CN increased, and the non-CKD participants with higher mtDNA-CN had a lower risk of developing CKD. These association between mtDNA-CN and CKD risk remained after adjusting for established risk factors, suggesting that lower mtDNA-CN may be an independent risk factor for CKD.

This study extends previous work on this topic in several ways. First, we considered various risk factors associated with CKD in older adults that may affect the correlation between mtDNA-CN and CKD. We found significant correlations between mtDNA-CN and age, and we adjusted for the effects of aging by controlling for age. And we controlled for the influence of inflammation on mtDNA-CN by considering the presence of white blood cells. The older adults always suffer from chronic diseases at the same time [26]. These diseases are important cause of CKD and may exacerbate the progression of CKD [27]. After controlling for these diseases, the association remained robust.

Second, there have been reports of lower mtDNA-CN being associate with higher risk of diabetes, metabolic syndrome and cardiovascular disease [27, 28]. Our subgroup analysis findings suggest that the association between mtDNA-CN and CKD is not influenced by the presence of diabetes. However, in contrast to our results, the He, et al. [16] found that this association was only present in patients with diabetes, and not in those without diabetes. Such differences could be related to differences in the populations, including variations in age, higher prevalence rates of diabetes and obesity compared to our study. Importantly, diabetes is not the primary cause of morbidity among older adults in the Chinese population [29]. These characteristics may affect the consistency of the results, as recently discussed by Kronenberg F, et al. [30].

Third, the association of mtDNA-CN with CKD was found to be comparable to that of established risk factors which change per 1-SD in our study, such as fasting glucose, diastolic blood pressure (DBP) and triglyceride (TG). This suggests a strong association between mtDNA-CN and CKD risk, emphasizing the importance of monitoring changes in mtDNA-CN. Fourth, we found that a higher mtDNA-CN was significantly associated with a reduced risk of kidney function declining to a worse stage, and the participants in higher mtDNA-CN had a lower risk of developing CKD. These findings suggest that mtDNA-CN could potentially serve as a useful biomarker for identifying CKD progression and severity, as well as a potential predictor for the development of CKD.

### Potential mechanism

The kidneys play a critical role in maintaining fluid balance, eliminating waste, and metabolite regulation, all of which require an ample supply of energy from mitochondria to sustain normal cellular metabolism and function [31]. As a very energy-hungry organ, many kidney cells particularly sensitive to mitochondrial dysfunction [32]. The entire protein-coding capacity of mtDNA is devoted to the synthesis of 13 essential subunits of the complexes of the respiratory apparatus [33]. Gene deficiencies lead to a cellular energy deficit, compromising kidney function [34]. By comparing CKD patients with healthy people, it has been found that CKD patients have an impaired mitochondrial respiratory system [6]. Moreover, the mitochondrial respiratory chain is a major source of reactive oxidative species (ROS) as byproducts of normal cell respiration. The respiratory chain function is impaired and thereby increase the electron leak in the respiratory chain and ROS production [27]. There is now an increasing body of evidence to suggest that the generation of ROS is significantly increased in kidney disease [35]. Further, the lack of proper mitochondrial integrity results in escape of its DNA content in cytosol, where it is recognized as “foreign” DNA. This recognition inappropriately activates the immune system, triggering pathological inflammation. The latest research published by Chung, et al. [36] confirmed this mechanism which lead to renal inflammation and fibrosis.

These large number of experimental studies clearly supports the findings of the serious renal pathology caused by the alteration of mtDNA-CN. Although there is unclear whether the causal relationship between mtDNA-CN and CKD [25]. In conjunction with observational associations with CKD adverse clinical outcomes and our ample evidence supporting a link between mitochondrial function and kidney disease progression in older, suggest that mtDNA-CN is a key factor in the early detection of CKD, and to monitor those expressing low mtDNA-CN with increased vigilance [37]. Moreover, mitochondria are closely related to healthy aging and longevity [38]. Recent studies have indicated that dietary supplements and exercise can enhance mitochondrial health by modifying mitochondrial function [39, 40]. Therefore, CKD patients, especially those with comorbidities, can improve mitochondrial function and disease management through lifestyle interventions such as adjusting corresponding dietary and exercise.

### Strengths and limitations

The present study had several strengths. On the one hand, it is one of the largest studies of mtDNA-CN and CKD in older adults, to ensure adequate statistical power to enable reliable evaluation. The population comes from the same region, living environment and other external factors rather homogeneous. On the other hand, the results were verified after testing for a variety of confounding factors that may influence CKD and mtDNA-CN in older adults. Therefore, we support the use of mtDNA-CN measurement in the whole blood as an easy-to-use biomarker for the risk stratification of older adults with CKD. The limitation of the present study is only had 2 years follow-up. Although there was a significant reduction in CKD incidence among individuals in the highest mtDNA-CN quartile, it is difficult to determine its predictive capacity.

## Conclusions

In conclusion, this study demonstrated that higher mtDNA-CN in peripheral blood is significantly associated with lower risk of CKD in older adults, independent of established CKD risk factors. This study in older adults adds to the growing body of evidence that mtDNA-CN plays an important role in kidney function.

### Electronic supplementary material

Below is the link to the electronic supplementary material.

Supplementary Material 1

## Data Availability

The health care data used in this study was retrieved from the primary medical center in Kunshan and anonymized for privacy purposes. Summary data that were used to support the findings of this study may be requested from the correspondent author.

## Abbreviations

CKD: chronic kidney disease
mtDNA-CN: mitochondrial DNA copy number
ICD-10: International Classification of Diseases-tenth version
BMI: body mass index
SBP: systolic blood pressure
DBP: diastolic blood pressure
eGFR: estimated glomerular filtration rate
SGPT: serum glutamic pyruvic transaminase
SGOT: serum glutamic-oxaloacetic transaminase
TC: total cholesterol
TG: triglyceride
HDL-C: high-density lipoprotein cholesterol
LDL-C: low-density lipoprotein
BUN: blood urea nitrogen
CCVD: Cardiovascular and cerebrovascular disease
