# Supplementary material for: Association of mitochondrial DNA copy number with chronic kidney disease in older adults
Source: BMC Geriatr. 2023 Aug 24;23:514. doi: 10.1186/s12877-023-04203-7 (PMC10463711; doi:10.1186/s12877-023-04203-7)
Supplement: Supplementary file 1 — Supplementary Material 1 [file 12877_2023_4203_MOESM1_ESM.docx]

# Supplementary data

## Table S1. The CKD patients were diagnosed by International Classification of Diseases-tenth version (ICD-10).

## Table S2. Results of the univariate logistic analysis for association of each covariate and CKD.

## Table S3. Sensitivity analysis of the association between mtDNA-CN and CKD prevalence in different comorbidity subgroups.

## Table S4. Sensitivity analysis of the association between mtDNA-CN and CKD prevalence in subgroups defined by different CKD definitions.

## Table S5. Analyze the phenotypic variable differences between individuals with CKD and those without CKD in the follow-up.

**Table S1.** The CKD patients were diagnosed by International Classification of Diseases-tenth version (ICD-10).

| **ICD-10** | **Description** |
| --- | --- |
| E11.2, E13.2 | diabetes mellitus with renal complications |
| I12, I13.1 | hypertensive renal disease |
| N03.x, N04.x, N05.x, R94.401 | glomerular disease |
| N11.9, N28.901 | renal tubulo-interstitial disease |
| N18.x, N19.x | renal failure |
| x is [arbitrary number](javascript:;) |  |

**Table S2.** The results of the univariate logistic regression analysis for the association of each variable with CKD.

| Variables | Univariable  Odds ratio (95% CI) | *P* value |
| --- | --- | --- |
| **mtDNA-CN** | **0.90 (0.87,0.93)** | **<0.001** |
| Hypertension | 2.45 (2.23,2.70) | <0.001 |
| CCVD | 2.30 (2.02,2.62) | <0.001 |
| Sex | 1.46 (1.36,1.57) | <0.001 |
| Diabetes | 1.44 (1.32,1.57) | <0.001 |
| Metabolic syndrome | 1.17 (1.07,1.28) | <0.001 |
| TG | 1.13 (1.08,1.17) | <0.001 |
| Age | 1.15 (1.14,1.16) | <0.001 |
| Fasting glucose | 1.09 (1.05,1.13) | <0.001 |
| SGOT | 1.07 (1.03,1.11) | <0.001 |
| SBP | 1.05 (1.02,1.09) | 0.005 |
| SGPT | 0.93 (0.86,0.97) | <0.001 |
| DBP | 0.86 (0.83,0.89) | <0.001 |
| TC | 1.04 (0.99,1.07) | 0.056 |
| HDL-C | 0.98 (0.95,1.02) | 0.28 |
| LDL-C | 1.01 (0.97,1.05) | 0.58 |
| BMI | 1.01 (0.97,1.05) | 0.47 |
| Waist | 1.01 (0.97,1.04) | 0.79 |

BMI: body mass index; SBP: systolic blood pressure; DBP: diastolic blood pressure; eGFR: estimated glomerular filtration rate; SGPT: serum glutamic pyruvic transaminase; SGOT: serum glutamic-oxaloacetic transaminase; TC: total cholesterol; TG: triglyceride; HDL-C: high-density lipoprotein cholesterol; LDL-C: low-density lipoprotein; BUN: blood urea nitrogen; CCVD: Cardiovascular and cerebrovascular diseases.

| mtDNA-CN | N/n (%) | Odds ratio (95% CI) | | *P* value | *P* for interaction |
| --- | --- | --- | --- | --- | --- |
|  |  | Univariable | Multivariable |  |  |
| **Total** | 3831/14467 (26.5) | 0.90 (0.87,0.93) | 0.90 (0.86,0.93) | <0.001 |  |
| **Diabetes** |  |  |  |  | 0.91 |
| yes | 1023/3172 (32.3) | 0.92 (0.85,0.99) | 0.91 (0.84,0.99) | 0.022 |  |
| no | 2808/11295 (24.9) | 0.90 (0.86,0.94) | 0.89 (0.84,0.93) | <0.001 |  |
| **Hypertension** |  |  |  |  | 0.29 |
| yes | 3233/10552 (30.6) | 0.90 (0.86,0.94) | 0.89 (0.85,0.93) | <0.001 |  |
| no | 598/3915 (15.3) | 0.95 (0.87,1.04) | 0.91 (0.83,1.00) | 0.063 |  |
| **Metabolic syndrome** |  |  |  |  | 0.35 |
| yes | 902/3121 (28.9) | 0.90 (0.83,0.97) | 0.93 (0.85,1.02) | 0.12 |  |
| no | 2929/11345 (25.8) | 0.90 (0.87,0.94) | 0.88 (0.84,0.92) | <0.001 |  |
| **CCVD** |  |  |  |  | 0.49 |
| yes | 447/1025 (43.6) | 0.97 (0.86,1.10) | 0.93 (0.81,1.07) | 0.29 |  |
| no | 3384/13442 (25.2) | 0.90 (0.86,0.94) | 0.89 (0.85,0.93) | <0.001 |  |

**Table S3.** Sensitivity analysis of the association between mtDNA-CN and CKD prevalence in different comorbidity subgroups

The multivariate model adjusted covariant are the same as the previous analysis, and the diseases used for stratification are excluded from each model. *P* for interaction < 0.05 is significant.

**Table S4.** Sensitivity analysis of the association between mtDNA-CN and CKD prevalence in subgroups defined by different CKD definitions.

|  |  | Odds Ratio (95% CI) | |  |
| --- | --- | --- | --- | --- |
|  | N/n (%) | Univariable | Multivariable | *P* value |
| **CKD_ICD_** | 578/3831 (15.1) | 0.87 (0.80,0.94) | 0.88 (0.81,0.96) | 0.005 |
| **CKD_eGFR_** | 3488/3831 (91.0) | 0.91 (0.87,0.95) | 0.90 (0.86,0.94) | <0.001 |

The part of CKD comes from ICD-10 as CKD_ICD_, the other part comes from the eGFR < 60 mL/min/1.73 m^2^ as CKD_eGFR_. The non-CKD participants as reference.

**Table S5.** Analyze the phenotypic variable differences between individuals with CKD and those without CKD in the follow-up.

| Characteristics | Follow Non-CKD | Follow CKD | *P* value |
| --- | --- | --- | --- |
|  | N=7,270 | N=230 |  |
| mtDNA-CN^*^ | 0.03 (0.98) | -0.10 (0.89) | 0.042 |
| **Demographic** |  |  |  |
| Age (years) | 69.0 [66.0, 73.0] | 72.0 [68.0, 77.0] | <0.001 |
| Sex = female (%) | 3634 (50.0) | 130 ( 56.5) | 0.059 |
| BMI (kg/m^2^) | 24.2 [22.2, 26.4] | 24.6 [22.9, 26.6] | 0.024 |
| Waist (cm) | 86.0 [80.0, 92.0] | 85.0 [80.0, 92.0] | 0.71 |
| **Laboratory** |  |  |  |
| SBP (mmHg) | 141.0 [129.0, 155.0] | 144.0 [132.0, 158.0] | 0.022 |
| DBP (mmHg) | 79.0 [72.0, 87.0] | 79.5 [72.2, 86.0] | 0.55 |
| SGPT (U/L) | 17.0 [13.0, 23.0] | 16.5 [13.0, 23.0] | 0.49 |
| SGOT (U/L) | 21.0 [18.0, 25.0] | 20.0 [16.0, 24.0] | <0.001 |
| TC (mmol/L) | 4.7 [4.1, 5.4] | 4.7 [4.1, 5.4] | 0.73 |
| TG (mmol/L) | 1.3 [1.0, 1.8] | 1.3 [1.0, 1.8] | 0.51 |
| HDL-C (mmol/L) | 1.3 [1.1, 1.5] | 1.3 [1.10, 1.5] | 0.32 |
| LDL-C (mmol/L) | 2.7 [2.2, 3.2] | 2.8 [2.3, 3.4] | 0.038 |
| Fasting glucose (mmol/L) | 5.4 [4.9, 6.1] | 5.6 [5.1, 6.8] | <0.001 |
| Creatinine (μmol/L) | 69.6 [61.0, 79.4] | 77.7 [70.4, 89.3] | <0.001 |
| eGFR (mL/min/1.73 m^2^) | 86.2 [77.4, 91.4] | 70.9 [65.4, 80.2] | <0.001 |
| BUN (mmol/L) | 5.4 [4.6, 6.4] | 6.1 [5.1, 7.1] | <0.001 |
| Uric acid (μmol/L) | 307.0 [257.4, 362.8] | 343.2 [288.2, 414.5] | <0.001 |
| **Comorbidities** |  |  |  |
| Diabetes =1 (%) | 1257 (17.3) | 91 ( 39.6) | <0.001 |
| Hypertension =1 (%) | 4957 (68.2) | 197 ( 85.7) | <0.001 |
| Metabolic syndrome =1 (%) | 1502 (20.7) | 59 ( 25.7) | 0.08 |
| CCVD =1 (%) | 315 ( 4.3) | 32 ( 13.9) | <0.001 |
| **Follow time (days)** | 464.0 [445.0, 485.0] | 368.0 [343.0, 405.2] | <0.001 |

BMI: body mass index; SBP: systolic blood pressure; DBP: diastolic blood pressure; eGFR: estimated glomerular filtration rate; SGPT: serum glutamic pyruvic transaminase; SGOT: serum glutamic-oxaloacetic transaminase; TC, total cholesterol; TG, triglyceride; HDL-C: high-density lipoprotein cholesterol; LDL-C: low-density lipoprotein; BUN: blood urea nitrogen; CCVD: Cardiovascular and cerebrovascular disease.

Data presented as median and interquartile range or number (%), unless otherwise indicated.

^*^ Mean and standard deviation.
